# Rapid and Integrated Quality Assessment of Organic-Inorganic Composite Herbs by FTIR Spectroscopy—Global Chemical Fingerprints Identification and Multiple Marker Components Quantification of Indigo Naturalis (*Qing Dai*)

**DOI:** 10.3390/molecules23112743

**Published:** 2018-10-24

**Authors:** Meng Pan, Wenxuan Pei, Yixin Yao, Ling Dong, Jianbo Chen

**Affiliations:** 1 School of Life Sciences, Beijing University of Chinese Medicine, Beijing 102488, China; pamela@bucm.edu.cn (M.P.); pwx3yy@163.com (W.P.); 2 School of Chinese Materia Medica, Beijing University of Chinese Medicine, Beijing 102488, China; 3 Kangmei Pharmaceutical Co., Ltd., Puning 515300, China; 14759155387@163.com

**Keywords:** indigo naturalis, quality assessment, ATR-FTIR spectroscopy, indigo, indirubin

## Abstract

This research aimed to develop an FTIR-based method for rapid and low-cost integrated quality assessment of organic-inorganic composite herbs, which are kinds of herbs composed of both organic and inorganic active ingredients or matrix components. A two-step quality assessment route was designed and verified using the example of Indigo Naturalis (*Qing Dai*). First, the FTIR spectra were used as global chemical fingerprints to identify the true and fake samples. Next, the contents of the organic and inorganic marker components were estimated by FTIR quantification models to assess the quality of the true samples. Using the above approaches, all the 56 true samples and five fake samples of Indigo Naturalis could be identified correctly by the correlation threshold of the FTIR chemical fingerprints. Furthermore, the FTIR calibration models provided an accurate estimation of the contents of marker components with respect to HPLC and inductively coupled plasma optical emission spectrometry (ICP-OES). The coefficients of determination (R^2^) for the independent validation of indigo, indirubin, and calcium were 0.977, 0.983, and 0.971, respectively. Meanwhile, the mean relative errors (MRE) for the independent validation of indigo, indirubin, and calcium were 2.2%, 2.4%, and 1.8%, respectively. In conclusion, this research shows the potential of FTIR spectroscopy for the rapid and integrated quality assessment of organic-inorganic composite herbs in both chemical fingerprints identification and marker components quantification.

## 1. Introduction

Unlike chemical and biological pharmaceuticals composed of defined active ingredients and excipients, the substantial and pharmacological foundations of herbal pharmaceuticals are usually not explicit enough to establish critical quality assessment criteria. For the time being, the contents of some marker components are often used as the expedient criteria to assess the quality of herbal pharmaceuticals. However, there may be a large number of unknown or undetermined active ingredients other than the marker components, which makes chemical fingerprints more and more popular to ensure consistency of herbal pharmaceuticals. As stated by the U.S. Pharmacopeia, chemical fingerprints are usually spectroscopic or chromatographic profiles for chemical identification by fingerprint comparison, while the fingerprinting methods should be capable of detecting as many constituents as possible [1]. The choice of the fingerprinting method is crucial for the quality assessment of herbal pharmaceuticals.

Besides the herbal materials with inherent inorganic crystals such as calcium oxalate and silica, there are kinds of organic-inorganic composite herbs containing considerable inorganic active ingredients or matrix components. A typical example is Indigo Naturalis (*Qing Dai*), which is a traditional Chinese medicinal used to counteract heat toxins and shows great potential for treating ulcerative colitis [2,3,4]. Indigo Naturalis is made from the leaves and stems of *Baphicacanthus cusia* (Nees) Bremek., *Polygonum tinctorium* Ait or *Isatis indigotica* Fort [5]. First, the fresh leaves with stems are immersed in water for several days to rot. Then, the stems are taken out and lime is added. The mixture is stirred and fermented to become purplish red. Finally, the foam on the liquid mixture surface is removed and dried to a powder. It is supposed that some precursor compounds in the original leaves and stems are transformed into indoles such as indigo and indirubin, while the particles of calcium carbonate and a few other inorganic components act as matrix carriers of organic molecules [6,7].

Although the pharmacological activities of indigo and indirubin have been proven [8,9], many other indole compounds and the rest components in Indigo Naturalis may also contribute to the therapeutic effects of this herb [4,10]. It should be mentioned that Indigo Naturalis is usually taken internally or applied topically in powder form [2,3,5], which means all organic and inorganic constituents are included. Therefore, the chemical fingerprints should be able to profile both organic and inorganic components to ensure the quality of Indigo Naturalis. However, the chromatographic methods cannot cover the inorganic components, while the element analysis methods cannot reflect the organic molecules. In this case, Fourier transform infrared (FTIR) spectroscopy is a potential fingerprinting method for Indigo Naturalis and other organic-inorganic composite herbs.

Being a direct and label-free analytical method, FTIR spectroscopy can detect both organic and inorganic molecules simultaneously [11,12,13]. In this way, FTIR spectra may be used as global chemical fingerprints of herbs. Furthermore, the contents of marker components may be estimated by FTIR spectra with the help of predefined calibration models [14,15]. That is to say, the single FTIR spectrum of an unknown herbal sample can implement integrated quality assessment procedures, including chemical fingerprints identification and multiple marker components quantification. Using convenient sampling techniques such as the attenuated total reflection (ATR) accessory, most herbal samples can be tested directly and quickly. Fingerprint comparison and marker components quantification can be performed automatically if pattern recognition and multivariate calibration models are available [16,17]. Taking the example of Indigo Naturalis, this research examines the feasibility of FTIR spectroscopy as a simple, rapid, cheap, and green method for the integrated quality assessment of organic-inorganic composite herbs.

## 2. Algorithm of Integrated Quality Assessment by FTIR Spectroscopy

**Figure 1:**
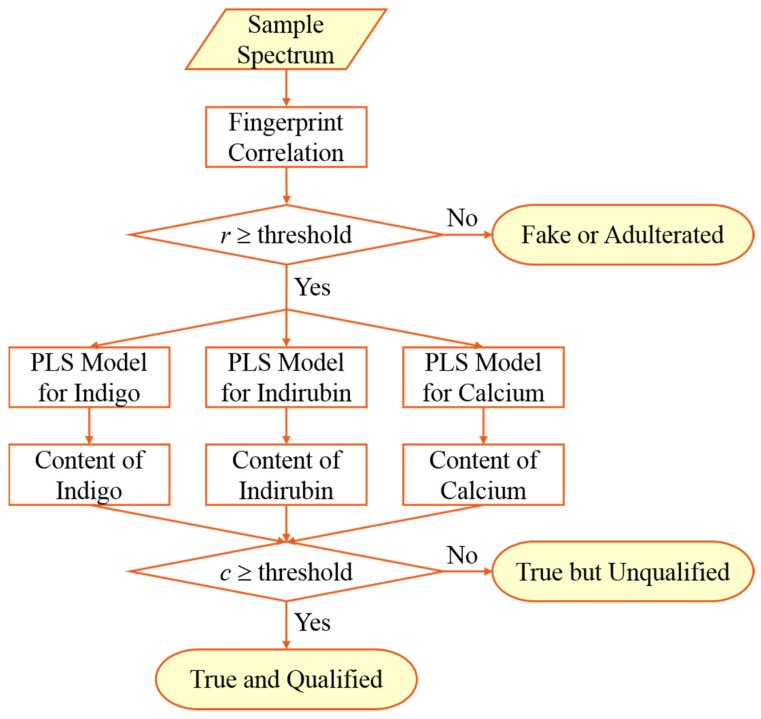
Algorithm of the integrated quality assessment of Indigo Naturalis by FTIR spectroscopy.

Identification is the first and foremost step in assuring the quality of herbal pharmaceuticals which should be made from defined materials. Therefore, the first step of integrated quality assessment of organic-inorganic composite herbs by ATR-FTIR spectroscopy is to identify the samples by comparing the spectral fingerprints against that of a reference. As the ATR-FTIR spectra are untargeted and global chemical fingerprints, the identification step is also an adulteration screening step. For true samples which can pass the fingerprint check, the contents of marker components should be determined for further quality assessment. As shown in Figure 1, correlation coefficients were used in this research to evaluate the similarity of the ATR-FTIR fingerprints of unknown samples against the reference spectrum of Indigo Naturalis, then the partial least squares (PLS) regression models were used to estimate the contents of indigo, indirubin, and calcium in the true samples.

## 3. Results and Discussion

### 3.1. Organic and Inorganic Marker Components of Indigo Naturalis

Indole compounds are supposed to be the main active ingredients of Indigo Naturalis [8,9,10]. The current Chinese Pharmacopeia states that the contents of indigo and indirubin in Indigo Naturalis should be not less than 2.0% and 0.13%, respectively [5]. As shown in Table 1, the content of indigo in the samples included in this research can be as low as 1.18%, and the lowest content of indirubin is 0.09%. In other words, there are many unqualified Indigo Naturalis samples on the market.

The main matrix of Indigo Naturalis particles should be calcium carbonate [6,7]. However, some manufacturers use the sediment rather than the foam of the fermented mixture to produce Indigo Naturalis containing complex inorganic impurities. Since all of the particles of Indigo Naturalis are usually taken internally or applied topically, the excess and unknown inorganic impurities can raise the risk of side effects or reduce the bioavailability of active ingredients. As a consequence, the content of calcium may be used for the first evaluation of the inorganic compositions of Indigo Naturalis to ensure quality and safety. As shown in Table 1, the normal samples of Indigo Naturalis contain more than 20% of calcium, which is much higher than the fake samples.

Although the primary quality assessment of Indigo Naturalis can be accomplished based on the contents of indigo, indirubin, and calcium, the classical measurement methods (such as HPLC and ICP-OES) of these marker ingredients require much time and effort. Therefore, FTIR spectroscopy is under consideration to develop a rapid method for the determination of organic and inorganic ingredients in Indigo Naturalis.

### 3.2. ATR-FTIR Spectral Characteristics of Indigo Naturalis

**Figure 2:**
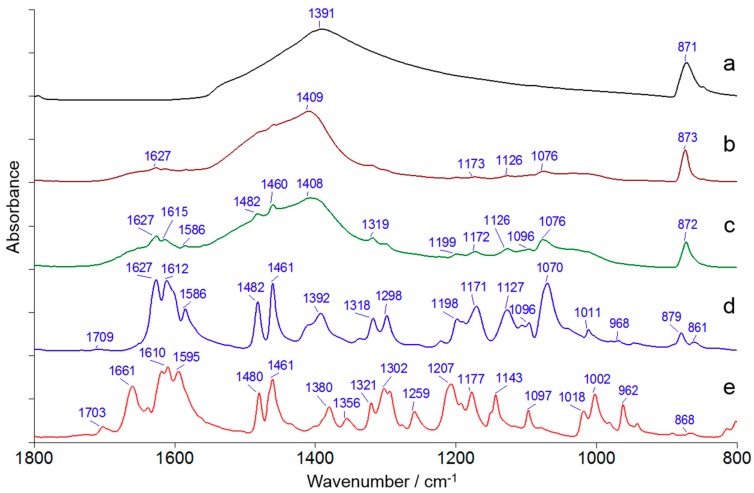
ATR-FTIR spectra of Indigo Naturalis and reference compounds. (a) CaCO_3_ (calcite); (b) sample T12; (c) sample T07; (d) indigo; (e) indirubin.

Figure 2 shows the ATR-FTIR spectra of typical samples of Indigo Naturalis and reference compounds. Since the main matrix of Indigo Naturalis is calcium carbonate, the feature bands of calcium carbonate near 1400 cm^−1^ and 870 cm^−1^ are prominent in the spectra of Indigo Naturalis samples [11]. As shown in Table 1, the content of indigo is usually an order of magnitude higher than that of indirubin in Indigo Naturalis. Besides, the molecular structures of indigo and indirubin are very similar. Consequently, the absorption bands of indirubin, which are weak and overlapped by the indigo bands, cannot be observed directly from the ATR-FTIR spectra of Indigo Naturalis.

The sample T07 contains 3.18% of indigo and 0.26% of indirubin, while the sample T12 contains 1.18% of indigo and 0.09% of indirubin. Accordingly, the sample T07 shows more characteristic peaks of indigo than the sample T12. As shown in Figure 2, with the peak threshold of 0.01A, as many as 11 peaks (1627, 1615, 1586, 1482, 1460, 1319, 1199, 1172, 1126, 1096, and 1076 cm^−1^) in the sample T07 can be assigned to indigo, while only 4 peaks (1627, 1173, 1126 and 1076 cm^−1^) of indigo can be observed in the sample T12.

Both inorganic and organic marker components of Indigo Naturalis can be observed in the ATR-FTIR spectra, but the manual comparison of characteristic peaks is complicated and inconsistent at times. Therefore, chemometrics tools are needed for the automatic and quantitative identification and determination of marker components of Indigo Naturalis by ATR-FTIR spectra.

### 3.3. Correlation-Based FTIR Chemical Fingerprint Identification of Indigo Naturalis

**Figure 3:**
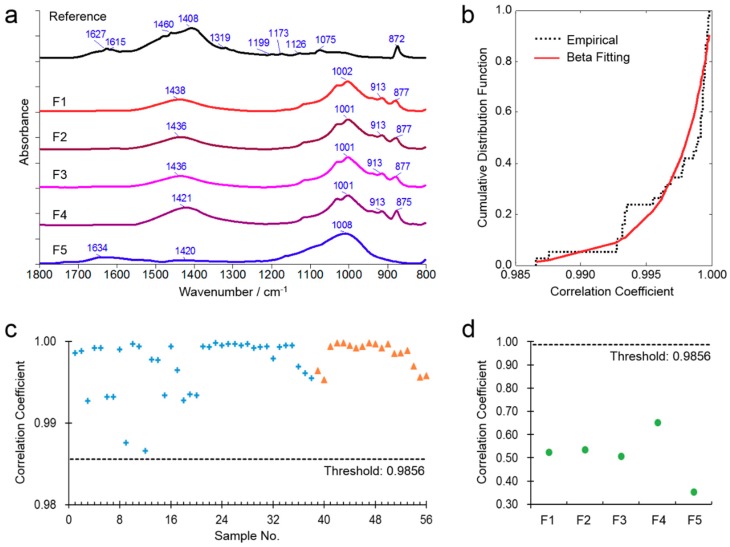
Correlation-based FTIR identification of Indigo Naturalis. (**a**) ATR-FTIR spectra of the reference and the fake samples F1–F5; (**b**) Cumulative distribution of the correlation coefficients of the true samples against the reference; (**c**) Correlation-based FTIR identification of the calibration true samples (+) and validation true samples (▲); (**d**) Correlation-based FTIR identification of the fake samples.

Since the chemical differences between the fake and true samples are usually larger than the differences among the true samples (Figure 3a), it is possible to discriminate the true and fake samples of Indigo Naturalis by the FTIR spectral similarities against the true reference. In order to establish the correlation threshold for the spectral fingerprints identification, the average ATR-FTIR spectrum of the 38 calibration samples of Indigo Naturalis in the region of 1800–800 cm^−1^ was used as the true reference spectrum.

As shown in Figure 3b, the beta distribution function was used to fit the cumulative distribution of the correlation coefficients of all calibration samples against the reference, and the lower distribution limit at the significant level of 0.05 was determined as the acceptance correlation threshold of the true samples. That is to say, a sample should be considered as true Indigo Naturalis if the correlation coefficients of this sample against the reference is above the acceptance correlation threshold, otherwise the sample may be fake or adulterated.

Based on the above rules and parameters, the ATR-FTIR acceptance correlation threshold of Indigo Naturalis was 0.9856. All calibration and validation samples passed the correlation check (Figure 3c), but all fake samples failed (Figure 3d). The results confirmed the sensitivity and specificity of the acceptance correlation threshold.

It should be pointed out that the correlation-based FTIR fingerprints identification method in this research is expected to distinguish the true and fake samples of Indigo Naturalis, not to assess good and poor samples. For this reason, all the true samples can pass the correlation check, no matter how much indigo or indirubin is contained in the samples.

### 3.4. Determination of Multiple Marker Components of Indigo Naturalis by FTIR Quantification Models

The contents of some marker components are needed for the further quality assessment of the true samples of Indigo Naturalis. Therefore, PLS quantification models based on the ATR-FTIR spectra were developed for the rapid estimation of indigo, indirubin, and calcium in Indigo Naturalis. There were 38 true samples included for the calibration of PLS models, while the remaining 18 true samples were employed for independent validation. The coefficient of determination (R^2^) and the mean relative error (MRE) were used to evaluate the model performance and optimize the model parameters. Finally, the ATR-FTIR spectra in the range of 1800–800 cm^−1^ after standard normal variate (SNV) normalization were used to develop the PLS models.

**Figure 4:**
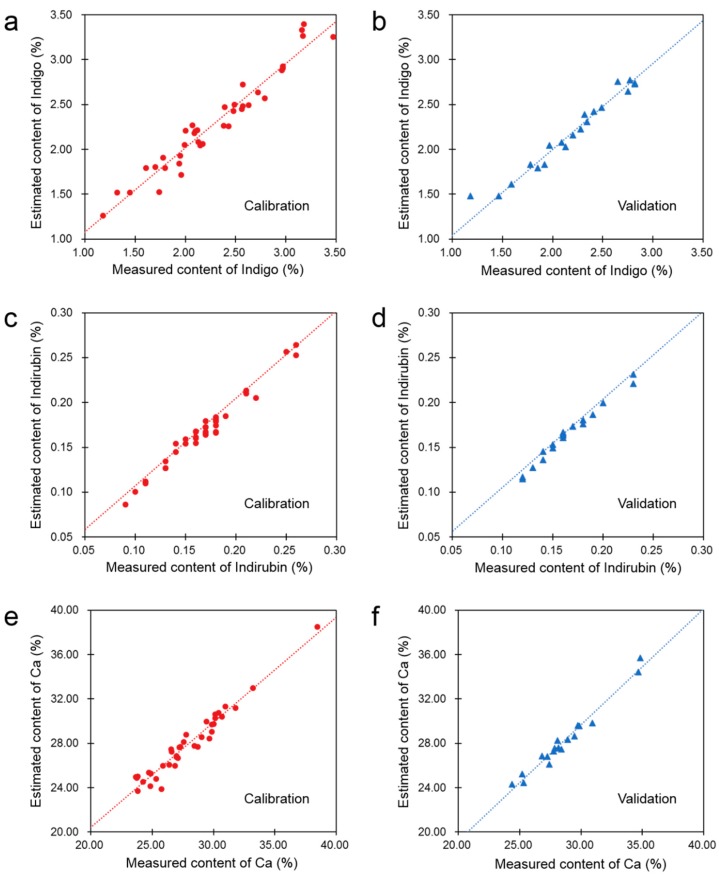
Comparisons between the ATR-FTIR model estimated and the reference method measured contents of the marker components of Indigo Naturalis. (**a**) Calibration samples (●) of the indigo model; (**b**) Validation samples (▲) of the indigo model; (**c**) Calibration samples (●) of the indirubin model; (**d**) Validation samples (▲) of the indirubin model; (**e**) Calibration samples (●) of the calcium model; (**f**) Validation samples (▲) of the calcium model.

As shown in Figure 4 and Table 2, the good linear relationships between the ATR-FTIR model estimated and the reference method measured contents of indigo, indirubin, and calcium of Indigo Naturalis can be observed in the calibration and validation samples. Besides, the mean relative error of the validation samples of each model was less than 3%. That is to say, the ATR-FTIR quantification models can provide quite good approximation of the organic and inorganic marker components in Indigo Naturalis.

## 4. Conclusions

This research shows the potential and the typical route of FTIR spectroscopy for the integrated quality assessment of organic-inorganic composite herbs such as Indigo Naturalis. The direct and label-free measurement approach makes FTIR a simple and rapid analytical technique. FTIR spectra can be used as global chemical fingerprints because the absorption signals of both organic and inorganic components can be observed simultaneously. With the help of some chemometrics tools, the fingerprints identification and multiple marker components quantification of a test sample can be completed automatically based on a single FTIR spectrum.

However, it should be noted that the sensitivity of FTIR spectroscopy is usually limited by the overlap of the absorption signals of different components in the same sample. In other words, the FTIR methods are good at rapid tests or exploring tests, rather than for precise tests of minor components in complex mixtures. Therefore, the FTIR methods proposed in this research are designed for the first and rapid quality assessment of organic-inorganic composite herbs. Further chromatographic, elemental and other measurements may be necessary for more detailed quality evaluations.

## 5. Material and Methods

### 5.1. Materials

A total of 61 Indigo Naturalis powder samples were collected from the markets. According to the abnormal dark blue color and the absence of indigo and indirubin, 5 fake samples were identified and labelled as F1–F5. All true samples of Indigo Naturalis were labelled as T01–T56, while 38 true samples were selected randomly as the calibration set and the remaining 18 true samples were assigned as the validation set to establish the fingerprint comparison and marker components quantification models.

Indigo (≥98.7%) and indirubin (≥99.0%) were purchased from China National Institutes for Food and Drug Control. Calcite (AR) was purchased from Sinopharm Chemical Reagent Co., Ltd (Shanghai, China). All reagents were used directly without any pretreatment.

### 5.2. Determination of Indigo and Indirubin by HPLC

**Figure 5:**
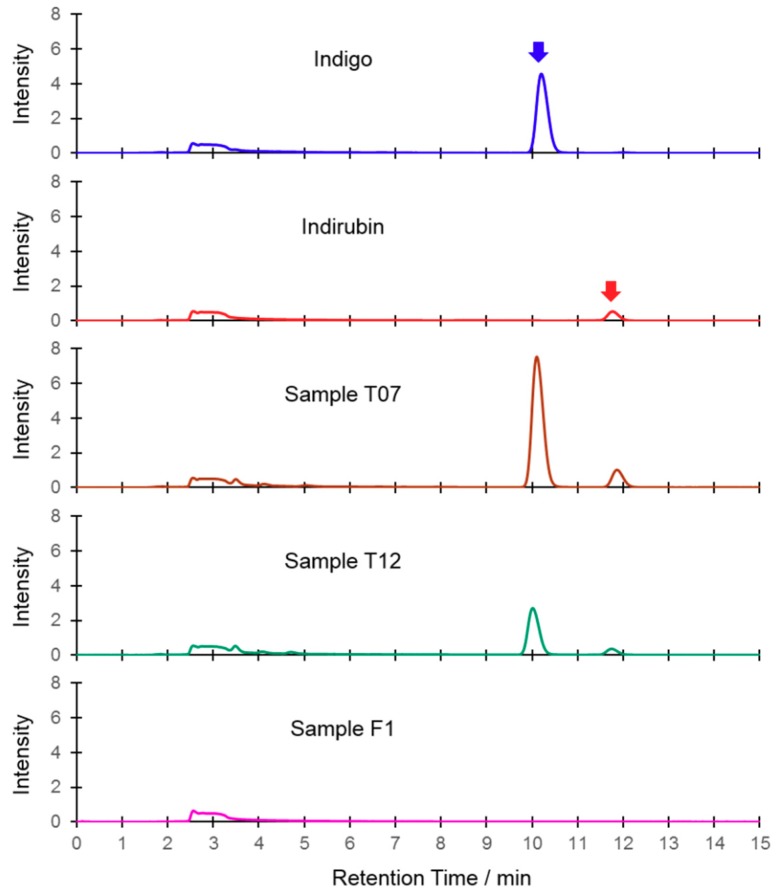
HPLC chromatograms of typical samples of Indigo Naturalis and the reference compounds.

Chromatographic determination of indigo and indirubin in Indigo Naturalis was performed using the LC-20AT HPLC system equipped with the SPD-20A UV detector and the SIL-20A autosampler (Shimadzu, Tokyo, Japan). A Kromasil C18 (250 mm × 4.6 mm, 5 µm) analytical column (Akzo Nobel, Bohus, Sweden) was used at 25 °C. The mobile phase was a mixture of acetonitrile (55%) and purified water (45%) with a flow rate of 1.0 mL/min. The detector wavelength was set at 290 nm. The injection volume was 20 µL. Duplicates of each sample were measured and the average value was used. Figure 5 shows the typical HPLC chromatograms of Indigo Naturalis and the reference compounds.

Preparation of sample solutions for quantification: the powder (0.05 g) of each sample was dispersed in 95 mL of *N*,*N*-Dimethylformamide (DMF), sonicated for 30 min, cooled to room temperature, and the final solution volume adjusted to 100 mL with DMF.

Preparation of standard solutions: indigo (5.98 mg) or indirubin (7.66 mg) was dispersed in DMF (95 mL), sonicated for 30 min, cooled to room temperature, and the final solution volume adjusted to 100 mL with DMF.

Development of the calibration curves: the standard solutions were diluted to a series of different concentrations and tested with the above HPLC conditions. Calibration curves were developed from the chromatographic peak area relative to the weights of indigo and indirubin. The calibration curves showed good linear relationships for indigo in the range of 0.0589–0.589 µg (R = 0.9996) and indirubin in the range of 0.00766–0.0766 µg (R = 0.9995).

### 5.3. Determination of Calcium by ICP-OES

Determination of calcium in Indigo Naturalis was performed using the VISTA-MPX ICP-OES spectrometer (Varian, USA). The radio frequency (RF) power was 1150 W. The flow rate of plasma gas, nebulizer gas, and auxiliary gas was 15 L/min, 0.75 L/min, and 1.5 L/min, respectively. The analytical line of Ca was 373.690 nm. The calibration curve was developed from Ca stock solution prepared from CaCO_3_.

The powder (0.1 g) of each sample was dissolved in aqua regia (6 mL), then 1 mL of hydrofluoric acid was added and the final solution volume was diluted to 100 mL. Duplicates of each sample were measured and the average value was used.

### 5.4. Measurement of ATR-FTIR Spectra

ATR-FTIR spectra of all powder samples were measured directly without any pretreatment. A Frontier FT-IR/NIR spectrometer with a universal ATR sampling accessory (PerkinElmer, Waltham, MA, USA) was used to collect the spectra. The internal reflection element (IRE) of the ATR accessory was a diamond/ZnSe composite crystal and the angle of incidence was 45°. The powder sample was placed on the top surface of the IRE crystal and pressed by a lever to ensure tight and consistent contact. Each spectrum was the average of 16 scans in the range of 4000–650 cm^−1^ with a spectral resolution of 4 cm^−1^. The influence of water vapor and carbon dioxide was subtracted automatically.

The software Spectrum v10.4.3 (PerkinElmer, USA) was used to control the spectrometer and process the spectra. First, the spectral ordinate was transformed into absorbance. Next, ATR correction with a contact factor of zero and automatic baseline correction were applied. Finally, the spectrum was normalized to set the maximum and minimum absorbance in the range of 1800–800 cm^−1^ at 1 and 0, respectively. Triplicates of each sample were measured and the average spectrum was used.

### 5.5. Calculations of ATR-FTIR Spectra

According to the ASTM standard [18], the spectral correlation coefficient is defined as the cosine of the angle between two spectrum vectors, which can be calculated as:

(1) $$r=\frac{\sum_{i=1}^{i=n}\left(x_{i}\times s_{i}\right)}{\sqrt{\sum_{i=1}^{i=n}x_{i}^{2}}\times \sqrt{\sum_{i=1}^{i=n}s_{i}^{2}}}$$

where *x_i_* and *s_i_* are the spectral intensities of the sample and the reference at the *i*th variable, and *n* is the number of spectral variables.

Since the spectral fingerprints reflect the chemical compositions, it is supposed that the true samples show higher spectral similarity than the fake or adulterated samples against the reference of Indigo Naturalis. Therefore, it is possible to define a correlation threshold to identify the unknown samples [19,20]. First, the average spectrum of the 38 calibration samples of Indigo Naturalis was used as the reference. Then, the correlation coefficients of all calibration samples against the reference were calculated. As the correlation coefficients were numerical values between 0 and 1, a beta distribution function was used to fit the correlation coefficients distribution and determine the acceptance threshold at a significant level of 0.05. Finally, the 18 validation samples of Indigo Naturalis and the 5 fake samples were used to examine the sensitivity and specificity of the correlation threshold. In this research, the above calculations were performed by the software MATLAB v7.0 (The MathWorks, Natick, MA, USA). ATR-FTIR spectra in the range of 1800–800 cm^−1^ were included after SNV normalization.

PLS regression models were developed with the software Spectrum Quant+ v4.6 (PerkinElmer, USA) for the quantitative estimation of indigo, indirubin, and calcium in the true samples of Indigo Naturalis. The model calibration and independent validation samples were the same as those used to determine the fingerprint correlation threshold. Parameters including the number of latent variables, the range of the spectra and the preprocessing methods were optimized. Finally, ATR-FTIR spectra in the range of 1800–800 cm^−1^ after SNV normalization were used to develop the PLS models. The coefficient of determination (R^2^) and the mean relative error (MRE) were used to evaluate the model performance, which were calculated as:

(2) $$R^{2}=1-\frac{\sum_{i=1}^{i=m}{\left(y_{i}-{\hat{y}}_{i}\right)}^{2}}{\sum_{i=1}^{i=m}{\left(y_{i}-\bar{y}\right)}^{2}}$$

(3) $$\mathrm{MRE}=\frac{1}{m}\times \overset{i=m}{\underset{i=1}{\sum }}\left|\frac{y_{i}-{\hat{y}}_{i}}{y_{i}}\times 100\%\right|$$

where $y_{i}$ and $\hat{y_{i}}$ are the specified and PLS model estimated content of the marker component in the sample *i*, respectively, $\bar{y}$ is the average value of the specified contents of the marker component in all samples, and *m* is the number of samples.

## Figures and Tables

**Table 1:** Contents of some marker components in true and fake Indigo Naturalis.

Ingredient (wt%)	True Samples			Fake Samples				
	Mean ± Std	Max	Min	F1	F2	F3	F4	F5
Indigo	2.28 ± 0.51	3.47	1.18	N.D.	N.D.	N.D.	N.D.	N.D.
Indirubin	0.17 ± 0.04	0.26	0.09	N.D.	N.D.	N.D.	N.D.	N.D.
Calcium	28.11 ± 2.97	38.47	23.65	7.01	7.10	6.97	10.91	1.47

N.D.: Not detected by the HPLC method used in this research.

**Table 2:** Performance of the ATR-FTIR quantification models of Indigo Naturalis.

Ingredient (wt%)	Indigo		Indirubin		Calcium	
	Calibration	Validation	Calibration	Validation	Calibration	Validation
Mean ± Std	2.29 ± 0.55	2.24 ± 0.43	0.17 ± 0.04	0.17 ± 0.03	27.86 ± 3.05	28.62 ± 2.80
Max	3.47	2.82	0.26	0.23	38.47	34.85
Min	1.18	1.46	0.09	0.12	23.65	24.37
Latent variables	6	6	10	10	9	9
R^2^	0.940	0.977	0.974	0.983	0.946	0.971
MRE	5.6%	2.4%	2.9%	2.2%	2.3%	1.8%
